# The effects of normalization on the correlation structure of microarray data

**DOI:** 10.1186/1471-2105-6-120

**Published:** 2005-05-16

**Authors:** Xing Qiu, Andrew I Brooks, Lev Klebanov, Andrei Yakovlev

**Affiliations:** 1 Department of Biostatistics and Computational Biology, University of Rochester, New York 14642, USA; 2 Functional Genomics Center, University of Rochester, 601 Elmwood Avenue, Rochester, New York 14642, USA; 3 Department of Probability and Statistics, Charles University, Sokolovska 83, Praha-8, CZ-18675, Czech Republic

## Abstract

**Background:**

Stochastic dependence between gene expression levels in microarray data is of critical importance for the methods of statistical inference that resort to pooling test-statistics across genes. It is frequently assumed that dependence between genes (or tests) is suffciently weak to justify the proposed methods of testing for differentially expressed genes. A potential impact of between-gene correlations on the performance of such methods has yet to be explored.

**Results:**

The paper presents a systematic study of correlation between the *t*-statistics associated with different genes. We report the effects of four different normalization methods using a large set of microarray data on childhood leukemia in addition to several sets of simulated data. Our findings help decipher the correlation structure of microarray data before and after the application of normalization procedures.

**Conclusion:**

A long-range correlation in microarray data manifests itself in thousands of genes that are heavily correlated with a given gene in terms of the associated *t*-statistics. By using normalization methods it is possible to significantly reduce correlation between the *t*-statistics computed for different genes. Normalization procedures affect both the true correlation, stemming from gene interactions, and the spurious correlation induced by random noise. When analyzing real world biological data sets, normalization procedures are unable to completely remove correlation between the test statistics. The long-range correlation structure also persists in normalized data.

## Background

There are two major methodological problems that deal with the issue of stochastic dependence between gene expression signals in microarray data. The first arises naturally when adjustments for multiplicity of tests are made by *pooling across genes (or tests) *in an effort to find differentially expressed genes in two-sample comparisons. The empirical Bayes methodology in the nonparametric [1-3] and parametric formulations [4,5], and closely related methods exploiting a two-component mixture model [6-8] represent typical examples. The common feature of such methods is that a test statistic (measure of differential expression) is first calculated for each gene to account for biological variability and then all the statistics (or the associated *p*-values) are pooled together and treated as a sample from which to estimate the sampling distribution of this statistic, the false discovery rate (FDR), q-values, etc. The same kind of pooling is typically used in maximum likelihood inference from microarray data [9,10] and some other methods of testing for differential expression of genes.

In all such approaches, the stochastic dependence between gene expression values or test statistics is a nuisance that hinders their application. The independence assumption is frequently invoked when building a theoretical foundation for a particular method of statistical inference. Some authors (e.g., [11]) allow for dependence between differentially expressed genes while assuming stochastic independence of those genes that do not change their expression between the two conditions under study. The biological rationale for such a hypothesis is unclear, because the normally functioning genes are involved in numerous biochemical pathways much like the altered ones.

The stochastic dependence between expression levels and thus between the associated test statistics is really a serious problem. It may cause high variability of statistical estimators and even deteriorate their consistency. To obtain theoretical results it is frequently assumed that weak or almost sure convergence holds for an empirical distribution function constructed from the data pooled across genes (see, i.e. [12,13]). However, this assumption is diffcult to validate biologically so that the required convergence to the true distribution function is always questionable; it may or may not be the case depending on the type and strength of stochastic dependence.

Storey [12] advocates the assumption of weak dependence between test-statistics when discussing some concerns raised in the paper by Ge, Dudoit, and Speed (hereafter abbreviated by GDS) [14]. It is worth quoting his line of reasoning at length:

"I hypothesize that the most likely form of dependence between the genes encountered in DNA microarrays is weak dependence, and more specifically, "clumpy dependence"; that is, the measurements on the genes are dependent in small groups, each group being independent of the others. There are two reasons that make clumpy dependence likely. The first is that genes tend to work in pathways, that is, small groups of genes interact to produce some overall process. This can involve just a few to 50 or more genes. This would lead to a clumpy dependence in the pathway-specific noise in the data. The second reason is that there tends to be cross-hybridization in DNA microarrays. In other words, the signals between two genes can cross because of molecular similarity at the sequence level. Cross-hybridization would only occur in small groups, and each group would be independent of the others."

This hypothesis does not seem plausible from a biological standpoint because of the pleiotropic character of gene function: one gene participates in multiple molecular pathways. However, the possibility that it may approximately be true for all practical purposes cannot be ruled out. There are two key words in the above quotation: "small groups" and "weak dependence". Whether or not such groups are small and stochastic dependence is suffciently weak can be deciphered only from real world data. To the best of our knowledge, no attempt has been made so far to systematically study dependence structures in microarray data using large data sets. In this connection we would like to continue quoting from [12]: "Many assumptions that have been made for modeling microarray data have yet to be verified. Hopefully evidence either for or against these assumptions will emerge... GDS have stressed the dependence between the genes... I leave it as a challenge to them to provide evidence from real microarray data that the aforementioned assumptions do not hold. I have not been able to find it myself". In the present paper, we take the first step in this direction by conducting an empirical study of the correlations between test statistics associated with different genes.

The second research area where the dependence between gene expression levels plays a crucial role is the discovery (reverse engineering) of molecular pathways and networks from microarray data [15]. A popular approach to pathway reconstruction is based on the sample correlation coeffcient or mutual information measures that are deemed to characterize interactions between genes via their products. These measures of interaction are computed from gene expression values observed across various experimental conditions. The snag here is that strong correlations in the raw (not normalized but background corrected) expression data may be induced by an array-specific technological noise, thereby producing numerous false-positive edges in the corresponding graph representing the underlying structure of a given pathway or network. However, if the data are normalized before the analysis, then the correlation structure of expression signals may be partially destroyed by the normalization procedure so that many edges in the resultant graph may be missing. The same applies to clustering techniques that utilize information on pairwise dependencies between the genes. The problem is less pressing where causal inference is possible from gene perturbation experiments. Although the present paper does not have a direct bearing on such settings, our results suggest that associative networks built on microarray data alone may have little to do with biological reality. The problem merits careful investigation in order to make the reverse engineering of this type more credible.

The present paper is focused on the correlations between test-statistics associated with expression signals produced by each gene and the effects of normalization procedures on these correlations. We limit our consideration to the *t*-statistic which is the most popular choice in microarray data analysis. Normalization is intended to mitigate the effect of technological noise that is inherent in microarray data. Normalization procedures tend to reduce the variability of original microarray data (Park *et al*, [16]), however no study has been carried out to assess the effect of such procedures on the correlation structure of microarray data in general and the correlation of *t*-statistics in particular. In a methodological study such as ours, it is a great advantage to have access to a large data set involving hundreds of arrays. We used the St. Jude Children's Research Hospital (SJCRH) Database on childhood leukemia which falls into this category. Computer simulations provide the necessary, albeit not very realistic, control where the actual model is known and arbitrarily large samples can be generated for testing various methodologies.

## Results

The design of our study is presented in the Methods section. This design allows us to compute the *t*-statistics (across arrays) for each gene and each pair of subsamples. This computation results in 15 values (corresponding to the 15 pairs of subsamples) of the *t*-statistic associated with each gene. Then we compute the sample correlation coeffcients between the *t*-statistics thus obtained for every pair of genes. The resulting coeffcients are summarized in the form of a histogram. We interpret such histograms as pertinent summary characteristics and not as estimators of some population distribution densities. We also look at pre-selected individual genes to determine the range of their correlation with all other genes. This range can be characterized by the number of gene pairs formed by a given gene with the correlation coeffcient exceeding some threshold level. We adopt the value of 0.5 as such a threshold.

Using these tools we attempt to answer the following questions:

• What is the (pairwise) correlation structure of the *t*-statistic in a large population of genes?

• What is the impact of normalization procedures on this structure?

• What is the impact of normalization procedures on the number of highly correlated pairs formed by a given gene?

**Figure 1:**
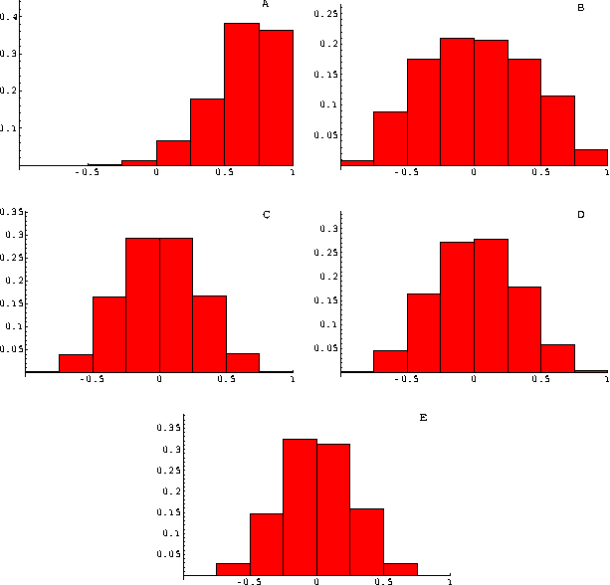
The histogram of correlation coeffcients for *overlapping *pairs of *t*-statistics associated with individual genes in the SJCRH data. A: data before normalization, B: *GEO*, C: *RANK*, D: *QUANT*, E: simulated set of data SIMU1.

Figure 1A shows the distribution (histogram) of correlation coeffcients for the *t*-statistics estimated from the SJCRH leukemia data for all pairs of genes. It is clear that the distribution is heavily shifted towards high positive correlation between the genes. In particular, more than 36% of pairs have their sample correlation coeffcients higher than 0.75 and only 7.6% have the coeffcients smaller than 0.25. The proportion of gene pairs with correlation coeffcients greater than 0.5 is 76%.

The effects of three normalization procedures (*GEO*, *RANK*, and *QUANT*, as defined in the Methods section) are shown in Figures 1B–1D. Figure 1E presents an ideal case where the *t*-statistics were obtained from independent normally distributed data (see the Methods section for explanations) produced by simulations (SIMU1). In this case, the proportion of gene pairs with correlation coeffcients greater than 0.5 is only 1.5%. While the normalization procedure *GEO *destroys a large proportion of correlation, the procedures *RANK *and *QUANT *outperform it as far as the reduction of between-gene dependence is concerned. The effects of the latter two procedures are largely similar. The procedure *RANK *reduces the proportion of correlation coeffcients greater than 0.5 to 4.3%, while the procedure *QUANT *reduces this proportion to 7.2%. For comparison, this indicator is equal to 14% for *GEO*. Thus the procedure *RANK *has the strongest effect on the correlation structure. Figure 1 in the Additional Material Files [see the file "Additional File 1"] shows essentially the same effect for randomly selected non-overlapping pairs of genes.

**Figure 2:**
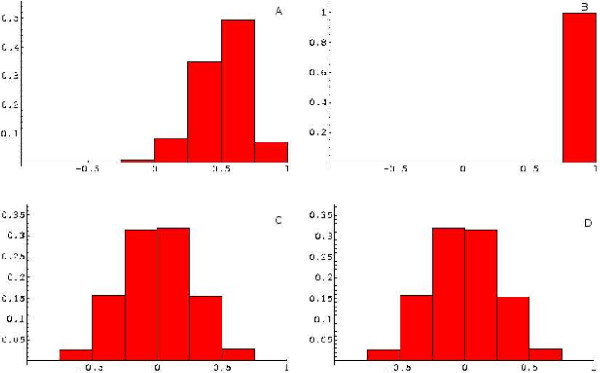
The effect of the normalization procedure *QUANT *as applied to the SIMU2N data. A: data without noise (SIMU2), B: data with noise (SIMU2N), C: SIMU2 after normalization, D: SIMU2N after normalization.

**Figure 3:**
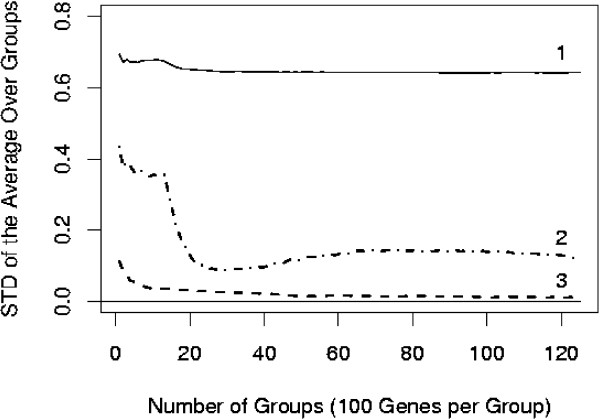
The behavior of the standard deviation of the sample mean as a function of the number of involved genes. 1. Raw biological data; 2. Quantile normalization; 3. Independent simulations (SIMU1).

The effect of normalization on the between-gene correlations observed in the simulated data SIMU2N and SIMU2 is stronger than that in the case of biological data (the SJCRH leukemia data set). This can be seen in Figures 2, 3, where only the results for the quantile normalization are shown. Again, if we look at the proportion of gene pairs with correlation coeffcients greater than 0.5, this indicator equals 1.5% for SIMU2N and 1.5% for SIMU2. The effects of *GEO *and *RANK *are displayed in Figures 1, 2 included in the Additional Material Files [see the file "Additional File 2"]. The stronger effect of *GEO *on the correlation structure of the SIMU2N data as compared to the SJCRH data comes as no surprise because the noise is simulated as an array-specific random effect, for which a heuristic justification of the *GEO *procedure is possible [20]. The normalization procedures exert their effect both on the correlation induced by the noise and on the true correlation that reflects interactions between gene products.

The effect of the quantile normalization for the SIMU3N, shown in Figure 3 in the Additional Material Files [see "Additional File 2"], deserves special discussion. Recall that each gene in the data set SIMU3N correlates only with a distinct group of genes termed a clump. Even if the genes involved in the same clump are heavily correlated, the average (over all pairs of genes) correlation coeffcient may still be quite low. When a uniformly distributed multiplicative random noise is imposed on each array, the genes pertaining to different clumps become highly correlated. The noise strengthens the intra-clump correlation as well. Recall that the clumpy structure of simulated data serves as a simplistic model of gene interactions within distinct pathways. As seen in Figure 3 [see "Additional File 2"], the normalization procedure *QUANT *is not nearly as effective as in the case of the SIMU2N data. This procedure does not eradicate the overall correlation between genes in the SIMU3N data. In this sense, the effects of normalization seen in the SIMU3N and in real biological data look similar.

Another way of studying such effects is to look at the number of pairs characterized by a relatively high correlation with a pre-selected gene. Tables 1, 2, 3 present the results for 20 genes that produce large numbers of highly correlated (with correlation coeffcients greater than 0.5). These initiator genes were identified through each of the data sets (simulated and biological) under study. The final column in every table gives the number of highly correlated pairs formed by a given gene before normalization. All the selected genes form such pairs with the overwhelming majority of genes. We term this type of dependence the long-range correlation. The number of highly correlated gene pairs remaining after a given normalization procedure serves as an indicator of its effciency.

**Table 1:** Long-range correlation analysis for the SIMU2N data.

Gene Label	GEO	QUANT	RANK	SIMU2N
1	743	746	741	12558
2	754	750	756	12558
3	723	723	721	12558
4	705	698	718	12558
5	736	734	754	12558
6	751	763	765	12558
7	702	695	709	12558
8	667	665	679	12558
9	747	747	759	12558
10	728	730	736	12558
11	713	717	713	12558
12	696	699	685	12558
13	743	750	762	12558
14	725	721	733	12558
15	691	691	740	12558
16	789	789	799	12558
17	724	725	669	12558
18	716	712	722	12558
19	762	762	720	12558
20	676	673	708	12558
Mean	724.6	724.5	729.5	12558
STD	30.1	31.8	31.9	0

**Table 2:** Long-range correlation analysis for the SIMU3N data.

Gene Label	GEO	QUANT	RANK	SIMU3N
1	483	520	512	12297
2	471	582	591	10656
3	436	523	614	12506
4	644	643	744	11031
5	677	739	765	11320
6	610	543	570	12413
7	612	863	788	12429
8	802	727	711	12077
9	1743	1406	1077	11898
10	975	895	920	12001
11	1352	1330	1543	12453
12	670	707	686	12480
13	1874	1849	1890	6913
14	1858	1765	1808	9371
15	1925	1790	1974	12469
16	1792	1718	1796	12520
17	1764	1526	1679	12499
18	1769	1684	1821	12509
19	1476	1300	1569	12514
20	2223	2307	2148	12507
Mean	1207.8	1170.9	1210.3	11743.2
STD	617.3	557.5	576.5	1402

**Table 3:** Long-range correlation analysis for the SJCRH data.

Gene Label	GEO	QUANT	RANK	raw data
1	5644	462	494	12481
2	7330	3175	1431	12486
3	4189	1480	2062	12496
4	5218	2728	1548	12493
5	8169	1888	1064	12451
6	8140	956	1162	12482
7	323	1169	839	12480
8	6774	1479	839	12497
9	7676	1832	2140	12390
10	8234	794	1440	12384
11	7652	930	466	12498
12	8266	1329	708	12476
13	8197	1343	2045	12391
14	7422	2118	2513	12501
15	1588	1467	1011	12494
16	7861	1931	1133	12429
17	1292	1477	1445	12489
18	6389	2949	1456	12481
19	7359	490	514	12469
20	4384	970	787	12488
Mean	6105.4	1548.4	1254.9	12467.8
STD	2545 2512	756	589.5	38.2

Consider first the results obtained with simulated data. Each of the twenty initiator genes selected from SIMU2N form exactly 12,558 highly correlated pairs. When applied to the SIMU2N data, the normalization procedures *RANK *and *QUANT *bring this number down to 700 on average (see Table 1). The variability in the size of this set of genes is low. For example, the number of highly correlated genes ranges from 661 to 794 after the application of the *QUANT *procedure. Both procedures indiscriminately reduce the true (intrinsic) correlation and its spurious (nuisance) counterpart. Although less effective, the procedure *GEO *does a similar job.

The results for the SIMU3N data are different (see Table 2). While the number of highly correlated gene pairs tends to decrease significantly for each of the twenty initiator genes, the size of this effect depends on the group of genes from which the initiator gene was chosen. This increases the variability of the number of highly correlated pairs remaining after normalization. For the *QUANT *method the range is from 526 to 2,368 showing that the remaining correlation extends far beyond the specified clumpy structure.

We then selected 20 initiator genes in the SJCRH data set representing real biological data. The number of highly correlated pairs formed by these genes before normalization ranges from 12,384 to 12,501, which is a very narrow range indeed. As is seen in Table 3, the procedure *GEO *does not destroy the correlation effectively; it leaves huge numbers (up to 8,266) of highly correlated gene pairs. The rank normalization results in much smaller numbers of highly correlated genes that range from the lowest of 494 to the highest of 2,513. The average is 1,255, which is about twice as much as we get from any normalized SIMU2N data. The variability is also very high, resembling a clumpy effect seen in the SIMU3N set. We do not consider this similarity as evidence for a clumpy structure of microarray data, but the results in Table 3 suggest that, if such a structure exists, an average clump should be expected to involve at least an order of magnitude more genes than the clump size postulated by Storey [12].

Another interesting finding in Table 3 is that the quantile normalization tends to leave more highly correlated genes in comparison to the rank normalization. This is contrary to our expectations based on the comparisons of correlation histograms reported above. The effect of the *QUANT *is also more variable than that of the *RANK*, which is another dissimilarity of practical importance. Leaving aside the fact that the *RANK *procedure is applied to gene expressions, while the *QUANT *works at the probe feature level, the difference between the two normalization methods is that we replace entries in an array by their ranks in the former case and by  in the latter. Recall that  is the average of entries having the same rank over all arrays. Obviously, the *QUANT *preserves more quantitative information in the data than does the *RANK *procedure. This explains why the result of the rank normalization is less variable.

The effect of the normalization *QUANT *on the distribution of the *t*-statistics across the genes for the actual and simulated data is shown in Figures 1, 2, 3, 4 included in the Additional Material Files [see the file "Additional File 3"]. From Figures 2 and 3, it is clear that, when applied to the simulated data SIMU2 and SIMU2N, this procedure makes the distribution of *t*-statistics similar to that in the ideal case shown in Figure 1 [see "Additional File 3"]. However, the effect appears to be somewhat less satisfactory with real data, especially in the tail regions of the resultant distribution of *t*-statistics.

The results shown in this section are obtained with a single initial random split of the pooled set of arrays into two groups. We have conducted several such splits in this study. All the above-described effects are highly reproducible, and reporting the results for other splits in the paper is not warranted.

## Discussion

It follows from our observations that normalization procedures are capable of destroying a significant part of correlations between gene expression signals and associated test-statistics. In doing so, they affect both the spurious correlation induced by the noise and the true correlation that reflects gene interactions. The clumpy structure (involving relatively large clumps of genes) of the SIMU3N data set is more resistant to this effect than the SIMU2N data. This is even more so for real biological data. The weaker effect of normalization seen in the SJCRH data indicates that the actual noise structure may be more complicated than assumed in the simulation studies (multiplicative array-specific random effect model). A clumpy structure of gene expression signals may also play a role in this phenomenon. This observation explains why it is so diffcult to remove correlations from the data.

The destructive effect of normalization procedures on pairwise correlations in microarray data is good news for the methods of statistical inference that resort to "pooling across genes". However, it remains unclear whether or not the remaining correlation may still be substantial enough to invalidate such methods by affecting important properties of statistical estimators and tests. The problem invites further investigation. However, we would like to present an experiment specially designed to address the consistency question mentioned in the Background section.

To this end, we applied the following algorithm to the SJRCH data:

1. Select randomly 100 genes and compute the arithmetic (sample) mean of the *t*-statistics across these genes for each pair of subsamples.

2. Compute the standard deviation of the sample mean across the 15 pairs of subsamples.

3. Select randomly 100 from the remaining genes and compute the arithmetic mean for the 200 genes for each pair of subsamples.

4. Compute the standard deviation from the sample means resulted from the previous step.

5. Continue until the set of all genes is exhausted.

6. Plot the estimated standard deviation of the sample mean as a function of the number of genes involved in each step of the algorithm.

7. Repeat the procedure *k *times to generate *k *trajectories of the standard deviation of the sample mean.

The results of one such experiment are given in Figure 3. It is known that the sample mean is an unbiased and consistent estimator for the true mean value in the case of independent and identically distributed observations. This case is represented by Curve 3 generated by simulations. It is clear that the standard deviation decreases very rapidly and tends to zero with increasing the number of genes. However, the same is not true for the biological data. For the raw data, the standard deviation does not show a distinct tendency to decrease (Curve 1). When the data are normalized using the quantile normalization procedure, the standard deviation first drops and then stabilizes at an approximately constant level, no matter how many (up to 12,500) genes are involved in its estimation (see Curve 2). This is clearly the effect of (long-range) correlation between the *t*-statistics associated with different genes. The pattern seen in Figure 3 was highly reproducible across *k *= 20 experiments with different random starts. If the standard deviation of an unbiased estimator tends to zero, this estimator is consistent. This is the case for Curve 3 but not quite so for Curve 2. While not a rigorous disproof of consistency of the sample mean in this case, the pattern seen in Curve 2 suggests that the estimator is likely to converge to a random variable (with the variance greater than zero) rather than to the true parameter to be estimated. This is definitely not a good sign for estimation procedures based on pooling across genes such as those built in the empirical Bayes methodology.

The observed effect of normalization procedures is definitely bad news for the associative network reconstruction from gene expression data. Unless further technological advancements result in a significant reduction of the noise in microarray data, this kind of analysis will continue producing unreliable inferences. To normalize, or not to normalize: that is the question to which no scientifically sound answer is currently known as far as this kind of reverse engineering is concerned. Although limited to cell cultures, the causal inference from gene perturbation (disruption and over-expression) experiments seems to be the only solid alternative. From this standpoint the observations reported in the present paper add to the concerns expressed by several investigators regarding how much confidence to place in the thousands of papers already published using microarray technology [17].

## Conclusion

The present paper provides quantitative insight into correlation between the *t*-statistics associated with different genes. This study leads us to conclude that:

• There is a long-range correlation in microarray data manifesting itself in a huge number of genes that are heavily correlated with a given gene in terms of the associated *t*-statistics.

• Using normalization of microarray data it is possible to significantly reduce correlation between the *t*-statistics computed for different genes.

• Normalization procedures affect both the true correlation, stemming from gene interactions, and the spurious correlation induced by random noise.

• It is likely that some noise effects represent non-monotone transformations of the underlying gene expression signals because even the rank normalization does not make the *t*-statistics independent when applied to the biological data.

• Even the most effcient normalization procedures are unable to completely remove correlation between the *t*-statistics associated with different genes in biological data. Furthermore, the long-range correlation structure persists in normalized data. This remaining correlation may be strong enough to deteriorate consistency of statistical estimators built from measurements on the genes.

## Methods

### Study design and biological data

There are 335 arrays (Affymetrix, Santa Clara, CA) in the SJCRH data set, each array representing *N *= 12, 558 genes. Each gene is represented in the data set by the logarithm of its expression level. The data are publicly available on the following website: [18]. The SJCRH data include the information on gene expression in normal blood and various types of childhood leukemia. The raw (background corrected but not normalized) expression data were generated by the output of the Bioconductor RMA (Robust Multi-Array Average) procedure when choosing the option: *normalization = false*. Since our focus was on purely methodological problems, we pooled all the available arrays together and shuffed the pooled sample. After randomly choosing and dropping 5 arrays (to make all subsamples of the same size), the pooled sample was randomly split into 30 parts, each containing 11 arrays.

Then 15 pairs of the array samples were arranged and the corresponding 15 *t*-statistics were computed for each gene, thereby mimicking 15 two-sample comparisons under the null hypothesis of no differential expression. As a result, each gene was associated with 15 values of the *t*-statistic so that the Pearson correlation coeffcient between the *t*-statistics thus derived could be computed for any pair of genes. The output of the above-described series of procedures is a 12, 558 × 15 matrix of *t*-statistics and the associated vector of correlation coeffcients for all pairs of genes. We proceeded through the same sequence of operations when analyzing normalized and simulated data sets.

In a separate experiment, we formed  non-overlapping pairs of genes to eliminate the spurious correlation between gene pairs due to multiple entries of the same gene in different pairs. Since this procedure begins with a randomly selected pair and proceeds through many steps of successive elimination of the previously selected pairs, it was repeated several times with different (random) starts and each output being analyzed separately. To study the long-range correlation, we picked 20 genes that produce large numbers of gene pairs with correlation coeffcients greater than 0.5. This experiment was designed to see how a given normalization procedure affects the number of such pairs associated with each of the twenty genes. A similar design was used with simulated data.

### Simulated data

We simulated several sets of data to gain a better insight into the effects of normalization. All of them included the same numbers of arrays and genes as in the biological data described in the previous section. Specific characteristics of these data sets are given below.

1. SIMU1: Every element *x*_*ij*_, *i *= 1, ..., 12, 558; *j *= 1, ..., 335 in SIMU1 represents log-intensity of expression of the *i*th gene from the *j*th array. The independent and identically distributed random variables *x*_*ij *_are generated from the standard normal distribution. This implies that the original expression signals are modeled as log-normally distributed random variables but we used their logarithms in our computations. This data set was used to illustrate the correlation analysis under independence of gene expression levels.

2. SIMU2 is a 12, 558 × 335 random matrix that models an exchangeable correlation structure. The entries in this matrix are normal random variables with mean zero and unit variance. The entries from different columns are independent, while the correlation coeffcient between any two elements *x*_*ij *_of the same column is equal to 0.8.

3. SIMU2N is a data set based on SIMU2. First we generate a 335-dimensional random vector *A*. The elements of *A *are independent and identically distributed. The marginal distri- bution of every element *a*_*ij *_of *A *is uniform over the interval [5,10]. This random vector is used to model an array-specific noise. We then define *y*_*ij *_to be *x*_*ij *_+ *a*_*j*_, where *x*_*ij *_is the *ij*-th entry in the SIMU2 data set. The new matrix *Y *= {*y*_*ij*_} represents the data SIMU2N

4. SIMU3 is a 12,550 × 335 matrix. The 12,550 rows (genes) are divided into ten groups of genes, each containing 1,255 rows. If two genes are both from the *k*-th group (gene numbers 100·(*k*-1)+1 through 100·*k*), for *k *= 1, 2, ..., 10, then the correlation coeffcient between them equals . Any two genes pertaining to different groups are stochastically independent.

5. SIMU3N is the same as the SIMU3 data set but with an added noise. An array-specific multiplicative and uniformly distributed noise is modeled exactly as in the SIMU2N data.

### Normalization methods

Suppose there are *M *arrays of length *N*, and we represent the corresponding log-intensities as an *N *× *M *matrix *X *such that each array is represented by a column in *X*. In this work, we used the following normalization methods:

#### 1. Geometric mean normalization GEO

If the array-specific random noise is multiplicative then a reasonable way to remove it from the expression values is to divide each element of the data matrix by the geometric mean over all gene expression signals on the array to which this element belongs. Szabo *et al *[20] discuss conditions under which this method is a valid one for testing two-sample hypotheses with microarray data.

#### 2. Rank normalization RANK

This method was proposed by Tsodikov *et al *[19] and discussed further in [20]. In accordance with their suggestion, we first obtain a vector *X*^*sort *^by arranging all gene expression signals for the same array in increasing order. Next we replace every entry in this array by its position (rank) in *X*^*sort *^counted from the smallest value. The idea behind this method is that ranks are invariant to any monotone transformation, implying a much more general model for the technological noise than the multiplicative array-specific random effect model.

#### 3. Quantile normalization QUANT

As discussed in [21,22], this method is motivated by the idea that a quantile-quantile plot shows that the distribution of *M *data vectors is the same if the plot is a straight line in the direction of unit vector  but it is not the same otherwise. So we could make a set of data to have the same distribution if we projected the points of an *M*-dimensional quantile plot onto the diagonal. Much like as with the *RANK *method, this approach is applied to genes rather than arrays. We refer the reader to [21,23] for more details. When working with the SJCRH data, this method was applied to probe feature level measurements. When working with simulated data, the method was applied to the levels of gene expression directly by processing them in exactly the same way.

## Authors' contributions

This work represents a truly collaborative endeavor. All members of the research team contributed equally to the design of this study, discussion of its technical issues and formulation of the net results. XQ was responsible for the computational component of the study. AB brought his biological expertise to the project.

## Supplementary Material

Additional File 1 The effect of normalization with non-overlapping pairs of genes; Click here for file

Additional File 2 The effect of normalization procedures on the correlation structure of simulated data; Click here for file

Additional File 3 The effect of the quantile normalization on the distribution of the *t*-statistics across genes. Click here for file
