# Supplementary material for: The effects of normalization on the correlation structure of microarray data
Source: BMC Bioinformatics. 2005 May 16;6:120. doi: 10.1186/1471-2105-6-120 (PMC1156869; doi:10.1186/1471-2105-6-120)
Supplement: Additional File 1 — The effect of normalization with non-overlapping pairs of genes; [file 1471-2105-6-120-S1.pdf]

Additional File 1.

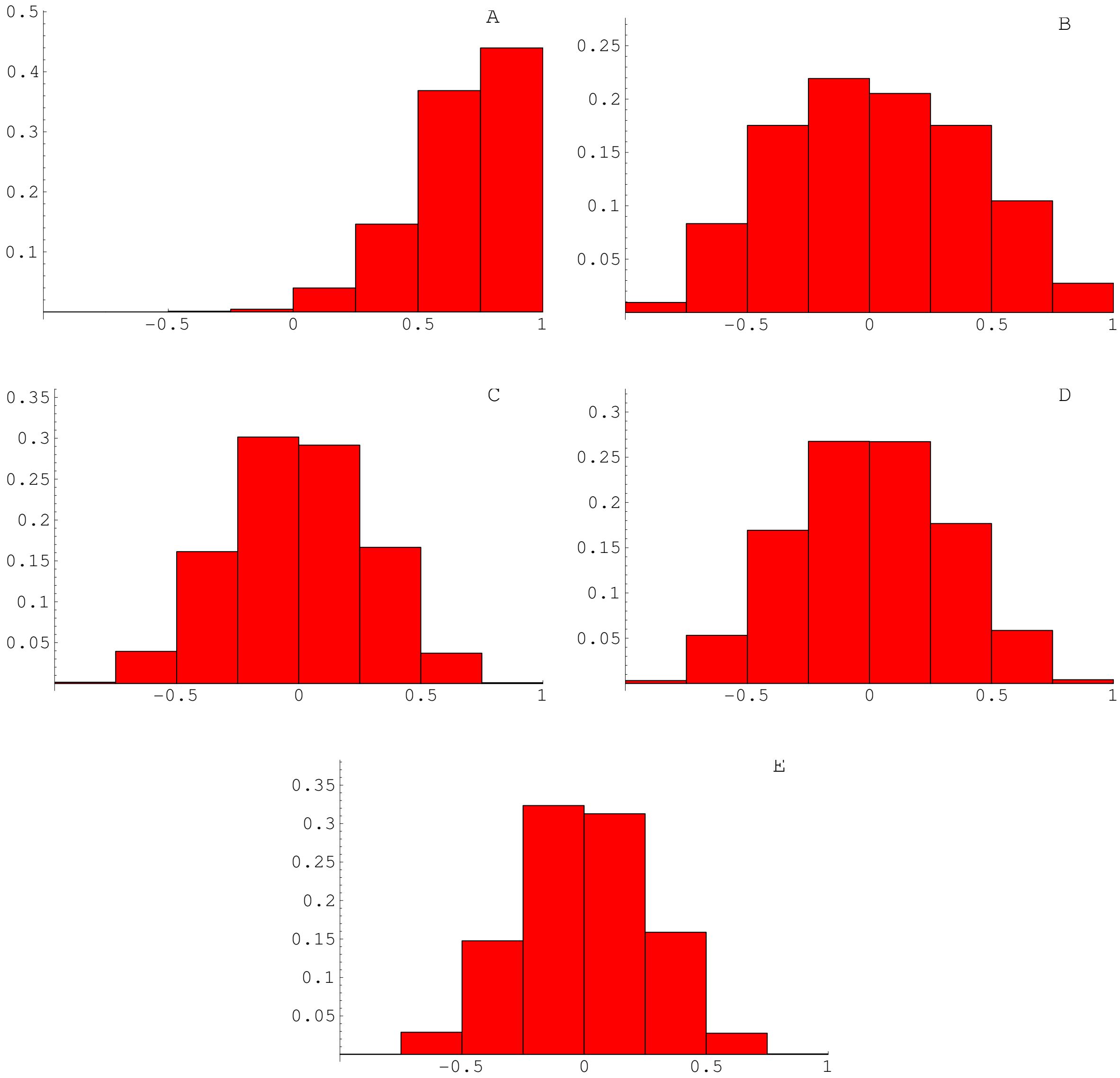

Figure 1: The histogram of correlation coefficients for *non-overlapping* pairs of t-statistics associated with individual genes in the SJCRH data. A: data before normalization, B: *GEO*, C: *RANK*, D: *QUANT*, E: simulated set of data SIMU1.
