# Supplementary material for: The effects of normalization on the correlation structure of microarray data
Source: BMC Bioinformatics. 2005 May 16;6:120. doi: 10.1186/1471-2105-6-120 (PMC1156869; doi:10.1186/1471-2105-6-120)
Supplement: Additional File 2 — The effect of normalization procedures on the correlation structure of simulated data; [file 1471-2105-6-120-S2.pdf]

# Additional File 2.

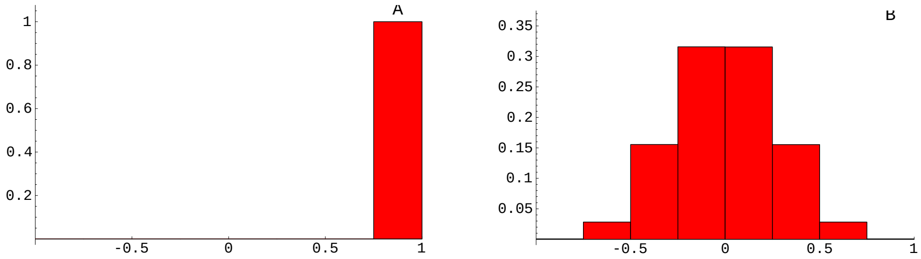


Figure 1: The effect of the normalization procedure *GEO* as applied to the SIMU2N data. A: before normalization, B: after normalization.

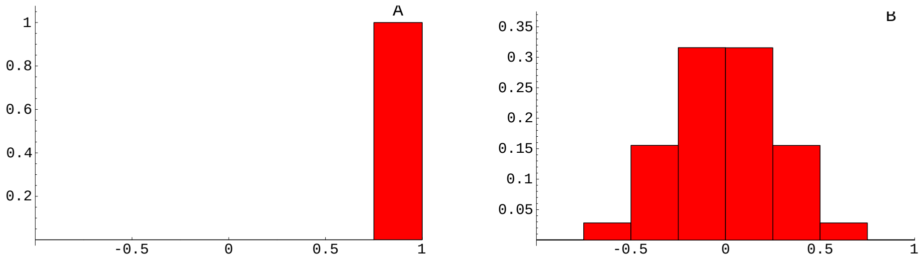


Figure 2: The effect of the normalization procedure *RANK* as applied to the SIMU2N data. A: before normalization, B: after normalization.

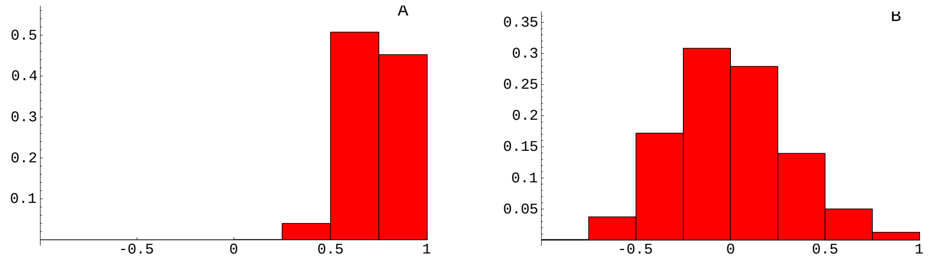


Figure 3: The effect of the normalization procedure $QUANT$ as applied to the SIMU3N data. A: before normalization, B: after normalization.
