# Supplementary material for: The effects of normalization on the correlation structure of microarray data
Source: BMC Bioinformatics. 2005 May 16;6:120. doi: 10.1186/1471-2105-6-120 (PMC1156869; doi:10.1186/1471-2105-6-120)
Supplement: Additional File 3 — The effect of the quantile normalization on the distribution of the t-statistics across genes. [file 1471-2105-6-120-S3.pdf]

Additional File 3.

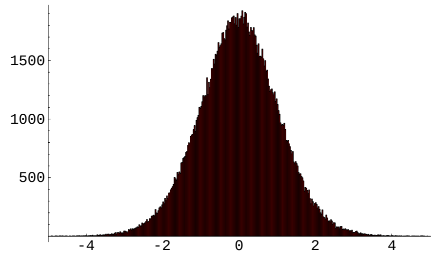

Figure 1: The distribution of the t-statistics in the case of independent and identically distributed data (SIMU1).

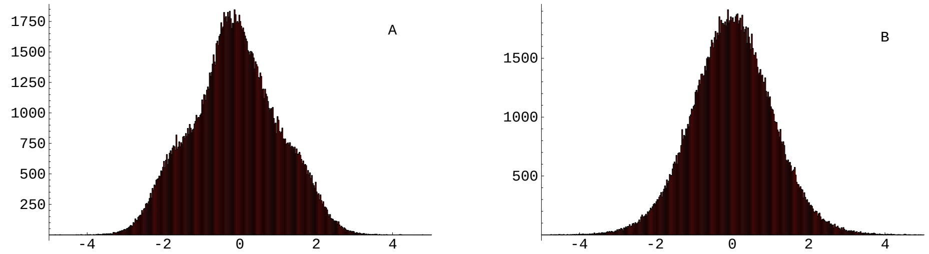

Figure 2: The effect of the normalization procedure $QUANT$ as applied to the SIMU2 data. A: before normalization, B: after normalization.

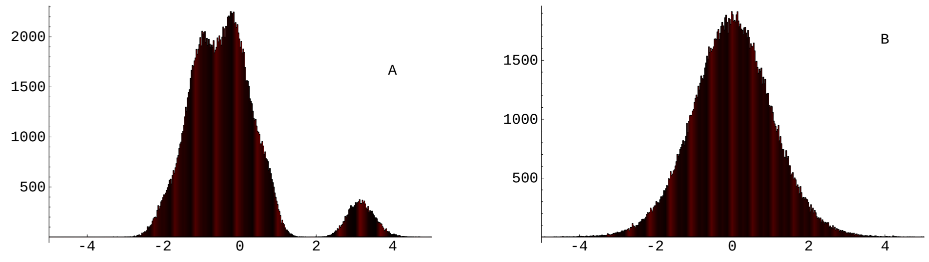

Figure 3: The effect of the normalization procedure *QUANT* as applied to the SIMU2N data. A: before normalization, B: after normalization.

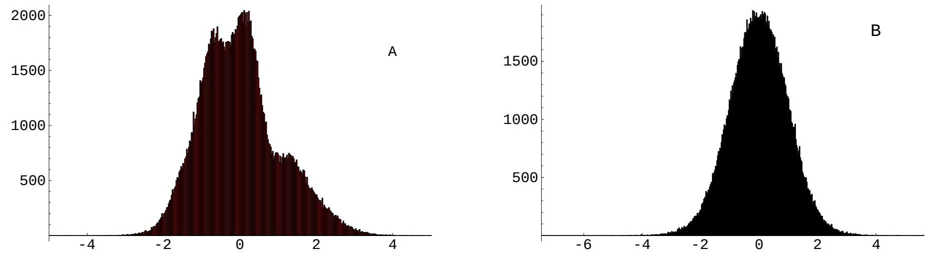

Figure 4: The effect of the normalization procedure *QUANT* as applied to the SJCRH data. A: before normalization, B: after normalization.
